# Associations of circulating proteins with lipoprotein profiles: proteomic analyses from the OmniHeart randomized trial and the Atherosclerosis Risk in Communities (ARIC) Study

**DOI:** 10.1186/s12014-023-09416-x

**Published:** 2023-07-03

**Authors:** Hyunju Kim, Alice H. Lichtenstein, Peter Ganz, Edgar R. Miller, Josef Coresh, Lawrence J. Appel, Casey M. Rebholz

**Affiliations:** 1 Department of Epidemiology, Johns Hopkins Bloomberg School of Public Health, 2024 East Monument Street, Suite 2-500, Baltimore, MD 21287 USA; 2 Welch Center for Prevention, Epidemiology, and Clinical Research, Johns Hopkins University, Baltimore, MD USA; 3 Jean Mayer USDA Human Nutrition Research Center on Aging, Tufts University, Boston, MA USA; 4 Department of Medicine, University of California San Francisco, San Francisco, CA USA; 5 Department of Medicine, Johns Hopkins School of Medicine, Baltimore, MD USA

**Keywords:** Nutrition, Nutrition/Carbohydrate, Nutrition/Protein, Proteomics, Lipoproteins, Clinical trials, Feeding study, Macronutrients, Dietary patterns

## Abstract

**Background:**

Within healthy dietary patterns, manipulation of the proportion of macronutrient can reduce CVD risk. However, the biological pathways underlying healthy diet-disease associations are poorly understood. Using an untargeted, large-scale proteomic profiling, we aimed to (1) identify proteins mediating the association between healthy dietary patterns varying in the proportion of macronutrient and lipoproteins, and (2) validate the associations between diet-related proteins and lipoproteins in the Atherosclerosis Risk in Communities (ARIC) Study.

**Methods:**

In 140 adults from the OmniHeart trial, a randomized, cross-over, controlled feeding study with 3 intervention periods (carbohydrate-rich; protein-rich; unsaturated fat-rich dietary patterns), 4,958 proteins were quantified at the end of each diet intervention period using an aptamer assay (SomaLogic). We assessed differences in log_2_-transformed proteins in 3 between-diet comparisons using paired t-tests, examined the associations between diet-related proteins and lipoproteins using linear regression, and identified proteins mediating these associations using a causal mediation analysis. Levels of diet-related proteins and lipoprotein associations were validated in the ARIC study (n = 11,201) using multivariable linear regression models, adjusting for important confounders.

**Results:**

Three between-diet comparisons identified 497 significantly different proteins (protein-rich vs. carbohydrate-rich = 18; unsaturated fat-rich vs. carbohydrate-rich = 335; protein-rich vs. unsaturated fat-rich dietary patterns = 398). Of these, 9 proteins [apolipoprotein M, afamin, collagen alpha-3(VI) chain, chitinase-3-like protein 1, inhibin beta A chain, palmitoleoyl-protein carboxylesterase NOTUM, cathelicidin antimicrobial peptide, guanylate-binding protein 2, COP9 signalosome complex subunit 7b] were positively associated with lipoproteins [high-density lipoprotein (HDL)-cholesterol (C) = 2; triglyceride = 5; non-HDL-C = 3; total cholesterol to HDL-C ratio = 1]. Another protein, sodium-coupled monocarboxylate transporter 1, was inversely associated with HDL-C and positively associated with total cholesterol to HDL-C ratio. The proportion of the association between diet and lipoproteins mediated by these 10 proteins ranged from 21 to 98%. All of the associations between diet-related proteins and lipoproteins were significant in the ARIC study, except for afamin.

**Conclusions:**

We identified proteins that mediate the association between healthy dietary patterns varying in macronutrients and lipoproteins in a randomized feeding study and an observational study.

**Trial registration:**

NCT00051350 at clinicaltrials.gov.

**Supplementary Information:**

The online version contains supplementary material available at 10.1186/s12014-023-09416-x.

## Background

Healthy dietary patterns have been recommended to prevent cardiovascular disease (CVD) [1, 2]. Within healthy dietary patterns, research has shown that manipulation of the proportion of macronutrient can reduce CVD risk [3]. Despite the evidence on healthy dietary patterns and macronutrient intake, key gaps in knowledge persist. The mechanisms through which macronutrients, within the framework of healthy dietary patterns, are associated with CVD is poorly understood.

Large-scale proteomic profiling offers an opportunity to address these gaps. Plasma proteins have diverse biological functions, including transporting nutrients and hormones, serving as enzymes and regulatory molecules, transmitting and receiving signals, and mediating immune responses [4–6]. These functions are relevant to digestion, absorption, transport, and metabolism of food. Thus, characterizing protein signatures of dietary patterns can expand our understanding of biological mechanisms.

Previous research on protein signatures of dietary patterns relied on self-reported dietary data, which has the limitation of potential systematic biases and measurement error [7–10]. Some of these prior studies reported that proteins associated with dietary patterns were involved in lipid metabolism [8–10], but no study investigated proteins that may mediate the association between dietary patterns and serum lipoprotein concentrations. Further, most studies did not validate significant associations in an independent population or in a different study design, making it challenging to distinguish between true and spurious associations.

The Optimal Macronutrient Intake Trial to Prevent Heart Disease (OmniHeart) was a randomized isocaloric feeding study consisting of 3 healthful dietary patterns which differed in the relative proportion of macronutrients (carbohydrate, protein, unsaturated fat) [3]. Compared to the carbohydrate-rich dietary pattern, the OmniHeart trial found that the protein-rich dietary pattern reduced low-density lipoprotein (LDL)-cholesterol (C), high-density lipoprotein (HDL)-C, triglycerides, and non-HDL-C (Additional file 1: Table S1). Compared to the carbohydrate-rich dietary pattern, the unsaturated fat-rich dietary pattern increased HDL-C, reduced triglycerides, and non-HDL-C, and had no significant effect on LDL-C concentrations. Compared to the unsaturated fat-rich dietary pattern, the protein-rich dietary pattern reduced HDL-C and triglyceride concentrations, and had no significant effect on LDL-C and non-HDL-C concentrations.

Using proteomics data generated from plasma specimens collected during a randomized feeding study (the OmniHeart trial), we aimed to determine whether any proteins mediate the association between dietary patterns and serum lipoprotein concentrations. Then, we aimed to validate the associations between diet-related proteins and lipoprotein outcomes in a large observational study [Atherosclerosis Risk in Communities (ARIC) study].

## Methods

### OmniHeart trial

#### Study design and study population

The OmniHeart trial was a randomized, cross-over, controlled feeding study with 3 intervention periods, which evaluated whether differences in macronutrient intake influences cardiovascular risk factors, specifically serum lipoprotein concentrations and blood pressure [3] (Additional file 1: Fig. S1). In two clinical sites (Baltimore, Maryland and Boston, Massachusetts), the trial recruited healthy men and women (≥ 30 years of age) with systolic blood pressure 120–159 mmHg, diastolic blood pressure 80–99 mmHg, LDL-C < 220 mg/dL (5.7 mmol/L), triglycerides < 750 mg/dL (8.5 mmol/L), and body weight < 350 lb (159 kg). Exclusion criteria included individuals who took blood pressure-lowering medication, lipid-lowering medication, or vitamin and mineral supplements, and those who drank > 14 alcohol beverage per week. The first participant began the trial in April 2003 and the last participant finished the trial in June 2005. Details on the study design and eligibility criteria are available elsewhere [3, 11]. Procedures were followed in accordance with the ethical standards of the institutional review boards (Johns Hopkins Medical Institutions and Brigham and Women’s Hospital) at the two sites and abide by the Declaration of Helsinki principles, and participants provided written informed consent.

Of the 164 participants, we excluded those with no plasma specimen (n = 3), proteomic data was unavailable for at least one of the diet interventions (n = 8), or a specimen for at least one of the diet interventions was flagged (n = 13) (Additional file 1: Fig. S2). Specimens were flagged by the manufacturer (SomaLogic, Boulder, Colorado) if they were determined to be of poor quality (e.g., calibration factors outside of the acceptable range). The final analytic sample was 140 participants.

### Controlled feeding interventions

During a 6 day run-in period, participants ate 2 days of meals from each of 3 dietary patterns [3]. After run-in, participants were randomized to receive 1 of 6 sequences of the 3 dietary patterns. Each diet sequence was followed by 14 to 18% of participants. Each diet phase was 6 weeks, with a 2–4 week washout period. Participants consumed their habitual diet during the washout periods. The original trial reported no evidence of differential carryover effects between dietary interventions [3]. All 3 diets were isocaloric, but varied in macronutrient content (Table 1) [11]. The carbohydrate-rich dietary pattern (58% carbohydrate, 15% protein, and 27% fat of total energy intake) was similar to the Dietary Approaches to Hypertension (DASH) dietary pattern. The protein-rich dietary pattern was comprised of 48% carbohydrate, 25% protein (half from plant sources such as grains, legumes, nuts and seeds, and soy), and 27% fat. The unsaturated-fat rich dietary pattern was comprised of 48% carbohydrate, 15% protein, and 37% fat, the majority (31%) of which came from mono- (21%) and polyunsaturated fat (10%). Sources of mono- and polyunsaturated fat included oils (olive, canola, and safflower), and nuts and seeds. All of the dietary patterns were similar to each other in that they were low in saturated fat, cholesterol, and sodium, and had high levels of fiber, potassium, calcium, and magnesium relative to a typical American diet. Details on study diets have been published [11].

**Table 1:** Macronutrient composition (% of energy) and selected micronutrient content of the study diets at 2,100 kcal in the OmniHeart trial^*a*^

	Carbohydrate-rich dietary pattern	Protein-rich dietary pattern	Unsaturated fat-rich dietary pattern
Carbohydrate	58	48	48
Protein	15	25	15
Plant sources^*b*^	5.5	12	5.5
Fat^*c*^	27	27	37
Saturated	6	6	6
Monounsaturated	13	13	21
Polyunsaturated fat	8	8	10
Cholesterol (mg)	150	150	150
Sodium (mg)	2300	2300	2300
Potassium (mg)	4673	4673	4673
Calcium (mg)	1240	1240	1240
Magnesium (mg)	497	497	497
Dietary fiber (g)	30	30	30

^*a*^ All 3 dietary patterns were isocaloric and were similarly healthful in that they were reduced in saturated fat, cholesterol, and sodium, and had higher levels of fiber, potassium, calcium, and magnesium relative to a typical American diet. Micronutrient targets and fiber (sodium, potassium, calcium, magnesium) are provided for the 2,100-kcal intake. The carbohydrate-rich dietary pattern was designed to be similar to the Dietary Approaches to Stop Hypertension (DASH) diet

^*b*^ Plant sources of protein include grains, legumes, nuts and seeds, and soy

^*c*^ Sources of mono- and polyunsaturated fat included oils (olive, canola, and safflower), and nuts and seeds

Participants’ body weight was monitored weekly. Total energy intake was adjusted to maintain participants’ body weight constant during the trial. All foods were provided by the study using standardized menus. Participants ate one meal onsite on a weekday, and received other foods packed for takeout. Participants were instructed to eat only the study foods provided and complete daily food diaries. In these diaries, participants recorded any study foods they missed or if they consumed non-study foods. Daily food diaries and observations during weekday visits were used to assess adherence. Protocol adherence was high; participants reported consuming all the study foods and not consuming non-study foods for 95% of person-days [3]. An objective biomarker of dietary intake, i.e., 24-hour urine excretion of urea nitrogen, demonstrated that protein intake was highest during the protein-rich dietary pattern intervention [3].

### Validation: Atherosclerosis Risk in Communities (ARIC) Study

#### Study design and study population

The ARIC study is a prospective cohort of 15,792 middle-aged adults (45–64 years of age). The study enrolled predominantly black and white men and women from four US communities (Forsyth County, North Carolina; Jackson, Mississippi; Minneapolis, Minnesota; Washington County, Maryland) in 1987–1989 (visit 1). Participants attended study visits periodically, from 1990 to 1992 (visit 2) to 2018–2019 (visit 7). Procedures were followed in accordance with the ethical standards of the Institutional Review Boards at all study sites, and participants provided informed consent.

For validation of diet-related proteins and lipoprotein outcomes, we used data from visit 2. At visit 2, the number of participants with proteomics data and lipoprotein outcomes was the highest. Of the 14,348 individuals who attended visit 2, we excluded non-black or non-white participants (n = 42, 32 Asian and 10 American Indian or Alaskan Native), and blacks in Minneapolis, Minnesota (n = 19) or blacks in Washington County, Maryland (n = 30) due to small numbers. Then we excluded participants with incomplete information on diet-related proteins (n = 2,537), or lipoprotein outcomes (n = 479), or covariates (n = 40) (Additional file 1: Fig. S3). The final analytic sample in the ARIC study was 11,201. After these exclusions, the proportion of black participants was 10.4% (n = 309) in Forsyth County, North Carolina (n = 2,958), and 100% (n = 2,303) in Jackson, Mississippi. In Minneapolis, Minnesota and Washington County, Maryland, all participants were white. Mean age (57 years) was similar across the four communities.

#### Covariates

At baseline (visit 1), participants reported age, sex, and race. At visit 2, participants reported smoking status, and trained personnel measured participants’ height and weight, which was used to calculate body mass index (BMI in kg/m^2^). At visit 2, serum creatinine was assessed using the modified kinetic Jaffe method, which was used to calculate kidney function [estimated glomerular filtration rate (eGFR)].

### Proteomic profiling in the OmniHeart trial and the ARIC Study

In the OmniHeart trial, fasting plasma specimens (8–12 h of fasting), which were stored at -70 °C since collection, were sent to SomaLogic for identification and relative quantification of proteins. Similarly, fasting plasma specimens (12 h) collected at visit 2 in the ARIC study were sent to SomaLogic. SomaLogic uses a Slow Off-rate Modified Aptamer (SOMAmer)-based capture tool named “SomaScan.” The SomaScan assay uses modified short single-stranded nucleotides to bind to specific epitopes, and can identify more than 5,000 proteins with high sensitivity. Details on the SomaScan assay have been reported previously and variability for inter- and intra-assay run was low (coefficients of variation ~ 5%) [12, 13]. Briefly, SOMAmers and plasma samples were mixed in 96-well plates. In the mixture, SOMAmer-protein complexes (cognate and non-cognate) formed, which were then captured on streptavidin beads. After washing away unbound proteins, the remaining protein complexes were labeled with biotin and were released from the beads using photocleavage and anionic competitor. As a result, non-cognate protein complexes dissociated, and this capture and release process was performed again to ensure specificity of SOMAmer-protein complexes (removing non-cognate proteins). The remaining SOMAmers were hybridized on a DNA microarray chip and abundance of each protein was expressed in units of relative fluorescence.

The SomaScan assay showed excellent reproducibility. We included 20 duplicates created from OmniHeart specimens for quality control, and 87% of proteins (4,599 out of 5,284) had Pearson correlation coefficients ≥ 0.8, and 97% proteins (5,093 out of 5,284) had coefficients of variation ≤ 20%. In the ARIC study, 625 duplicates created from visit 2 data showed high reproducibility (median Pearson correlation coefficient: 0.93, median coefficient of variation: 6.3%).

The SomaScan assay identified 5,284 proteins in the OmniHeart trial and the ARIC study, and a complete list of these proteins have been published in a prior study [14]. We excluded proteins bound to FC mouse (n = 228), proteins bound to contaminants (n = 15), non-proteins (n = 70), and proteins without a UniProt ID (n = 5). We additionally excluded proteins with coefficient of variation ≥ 50% (n = 6) or variance < 0.01 (n = 2) on the log scale. In total, 4,958 proteins were used for analyses in the OmniHeart trial. All proteins were log_2_-transformed to account for skewness, and outliers were capped at 5 standard deviations (SDs) above or below the mean. The same steps used in the OmniHeart trial were applied to the proteomics data in the ARIC study, including log_2_-transformation of all proteins. In the ARIC study, we focused only on the significant proteins from the OmniHeart trial.

### Lipoprotein outcomes in the OmniHeart trial and the ARIC study

All lipoprotein outcomes were assessed in serum in the OmniHeart trial and the ARIC Study. We focused on 5 lipoproteins: LDL-C, HDL-C, triglycerides, non-HDL-C, and the ratio of total cholesterol to HDL-C. We did not include total cholesterol in our analysis because the effect of the diet interventions varied for HDL-C and non-HDL-C [3]. Fasting serum samples were collected at the end of each diet intervention period, stored at -70 °C, and sent to the Core Laboratory for Clinical Studies (School of Medicine, Washington University, St Louis, Missouri) for analyses. HDL-C and triglycerides were assessed using an enzymatic assay (Thermo Scientific, Waltham, Massachusetts). Total cholesterol was measured using enzymatic methods on the Roche Hitachi 917 analyzer. LDL-C was estimated using the Friedewald equation [15]. Non-HDL-C was calculated as the difference between total cholesterol and HDL-C, and total cholesterol to HDL-C ratio was calculated by dividing total cholesterol by HDL-C.

In the ARIC study, fasting blood sample was used to assess lipoprotein concentrations at a central lipid laboratory (Baylor University, Houston, Texas). Triglycerides and total cholesterol were assessed using enzymatic methods (GPO Triglyceride and Monotest Cholesterol procedures, Boehringer Mannheim) [16]. HDL-C was assessed after precipitating non-HDL lipoproteins, and LDL-C was calculated using the Friedewald Eqs. [15, 17]. We used the same approaches to calculate non-HDL-C and total cholesterol to HDL-C ratio.

### Statistical analysis

We compared the baseline characteristics of the original OmniHeart trial participants (N = 164) and the analytic sample (N = 140) using proportions for categorical variables and means and SDs for continuous variables.

In the OmniHeart trial, we first identified diet-related proteins, using paired t-tests to evaluate differences in log_2_-transformed protein abundance in 3 different comparisons: (1) protein-rich vs. carbohydrate-rich dietary patterns (reference), (2) unsaturated fat-rich vs. carbohydrate-rich dietary patterns (reference), (3) protein-rich vs. unsaturated fat-rich dietary patterns (reference). We conducted 3 pairwise comparisons to be consistent with the comparisons from the original OmniHeart trial [3]. To account for multiple testing, we adjusted the threshold to assess statistical significance using the Bonferroni method (0.05/4,958 proteins/3 comparisons = 3.36 × 10^− 6^).

Then, we assessed whether differences in diet-related proteins from each diet comparison (exposures) were associated with differences in 5 lipoproteins (outcomes; LDL-C, HDL-C, triglycerides, non-HDL-C cholesterol, ratio of total cholesterol to HDL-C) using linear regression models. For each diet comparison, we adjusted the alpha level using the Bonferroni method based on the number of significant diet-related proteins [protein-rich vs. carbohydrate-rich dietary patterns: 5.56 × 10^− 4^ (0.05/18 diet-related proteins/5 lipoprotein outcomes); unsaturated fat-rich vs. carbohydrate-rich dietary patterns: 2.99 × 10^− 5^ (0.05/335 diet-related proteins/5 lipoprotein outcomes); protein-rich vs. unsaturated fat-rich dietary patterns: 2.51 × 10^− 5^ (0.05/398 diet-related proteins/5 lipoprotein outcomes)]. As an exploratory analysis, we conducted sex-stratified analyses among the significant proteins using the same linear regression models.

Next, we used causal mediation analysis to identify which, if any, proteins mediate the association between each diet comparison and lipoproteins (Additional file 1: Fig. S4). This approach produces estimates of direct effects of the exposure on the outcomes (effect of dietary patterns on lipoproteins with no mediation) and indirect effects (effect of dietary pattern on lipoproteins that is mediated by proteins) which accounts for the effect of mediators [18]. For mediation analysis, we fit two linear mixed models with random intercepts, which allows each participant to serve as their own control, consistent with the design of the OmniHeart trial. The first model evaluated the association between each diet comparison and diet-related proteins (mediator). The second model evaluated the association between each diet comparison and lipoproteins, after controlling for diet-related proteins. We used the “mediation” package in R and calculated the uncertainty with 1,000 bootstrapped samples. For each protein, we reported the proportion mediated (indirect effect divided by the sum of direct and indirect effects). We excluded one protein-lipoprotein association due to non-significant mediation (*P*-value > 0.05), and 2 proteins which produced negative proportion mediated. Negative proportion mediated may be due to inconsistent mediation which results from the mediated effect having an opposite direction of association from the direct effect [19].

Lastly, the associations between diet-related proteins and lipoprotein outcomes were validated in the ARIC Study using multivariable linear regression models. In this analysis, we used levels of diet-related proteins as the exposure variables and lipoprotein concentrations as the response variables (cross-sectional associations). We conducted multivariable linear regression models, adjusting for age, sex, a combined term of race and study center to account for unequal distribution of race across study centers, smoking status, BMI, and eGFR. Associations were considered validated at the Bonferroni threshold (0.05/13 diet-lipoprotein associations from the OmniHeart trial = 3.85 × 10^− 3^).

All analyses were conducted using R version 4.1.0 (R Foundation for Statistical Computing, Vienna, Austria).

## Results

### Baseline characteristics of the participants

Baseline characteristics of the original trial participants (N = 164) and the analytic sample (N = 140) were similar (Table 2). The mean age of the analytic sample was 53.7 years, compared to 53.1 years in the trial participants. Less than half of the analytic sample was composed of women, and more than half of the analytic sample consisted of African American participants, similar to the trial participants. The proportion of participants who were never smokers, current alcohol consumers, obese, and who had hypertension was similar in the analytic sample compared to the trial participants. Given such similarities, we did not adjust for covariates in subsequent analyses in the OmniHeart trial. In the ARIC Study, the mean age was 57 years, more than half of the participants was women, and 23% of the participants was African American. More than one-third of the ARIC Study participants were college graduates, never or former smokers, overweight, and were hypertensive.

**Table 2:** Baseline characteristics of participants in the original trial and analytic sample in the OmniHeart trial and the Atherosclerosis Risk in Communities (ARIC) Study^*a*^

	OmniHeart trial participants (N = 164)	OmniHeart analytic sample (N = 140)	ARIC Study analytic sample (N = 11,201)
Age, y	53.1 (10.8)	53.7 (10.3)	57.1 (5.7)
Women, n (%)	73 (44.5)	67 (47.9)	6223 (55.5)
African American, n (%)	90 (54.9)	83 (59.3)	2612 (23.3)
Education, n (%)			
High school graduate or less	33 (20.1)	29 (20.7)	2428 (21.7)
Some college	56 (34.1)	49 (35.0)	4709 (42.0)
College graduate	75 (45.7)	62 (44.3)	4049 (36.1)
Smoking, n (%)			
Current smoker	18 (11.0)	17 (12.1)	2492 (22.2)
Former smoker	46 (28.0)	39 (27.9)	4241 (37.9)
Never smoker	100 (61.0)	84 (60.0)	4468 (39.8)
Current alcohol consumer, n (%)	73 (44.5)	59 (42.1)	6361 (56.7)
Total energy intake, kcal	2315 (1174)	2256 (1153)	—
BMI, kg/m^2^	30.3 (6.1)	30.5 (5.8)	27.9 (5.3)
BMI category, n (%)			
Not overweight or obese	34 (20.7)	25 (17.9)	3490 (31.2)
Overweight	55 (33.5)	48 (34.3)	4479 (39.9)
Obese	75 (45.7)	67 (47.9)	3232 (28.9)
SBP, mm Hg	131.2 (9.4)	131.2 (9.6)	121.5 (18.8)
DBP, mm Hg	77.0 (8.2)	77.2 (8.4)	71.9 (10.3)
Hypertensive status, n (%)^*b*^	32 (20.0)	28 (20.0)	4026 (35.9)

^*a*^ Values are n (%) for categorical variables and mean (standard deviation) for continuous variables

^*b*^ Hypertensive status was defined as SBP ≥ 140 mm Hg or DBP ≥ 90 mmHg in the OmniHeart trial. In the ARIC Study, hypertensive status was defined as SBP ≥ 140 mm Hg, DBP ≥ 90 mmHg, or use of antihypertensive medication use in the past 2 weeks

— indicates that total energy intake was not assessed at ARIC visit 2

DBP, diastolic blood pressure; SBP, systolic blood pressure

### Proteins associated with dietary patterns

**Fig. 1:**
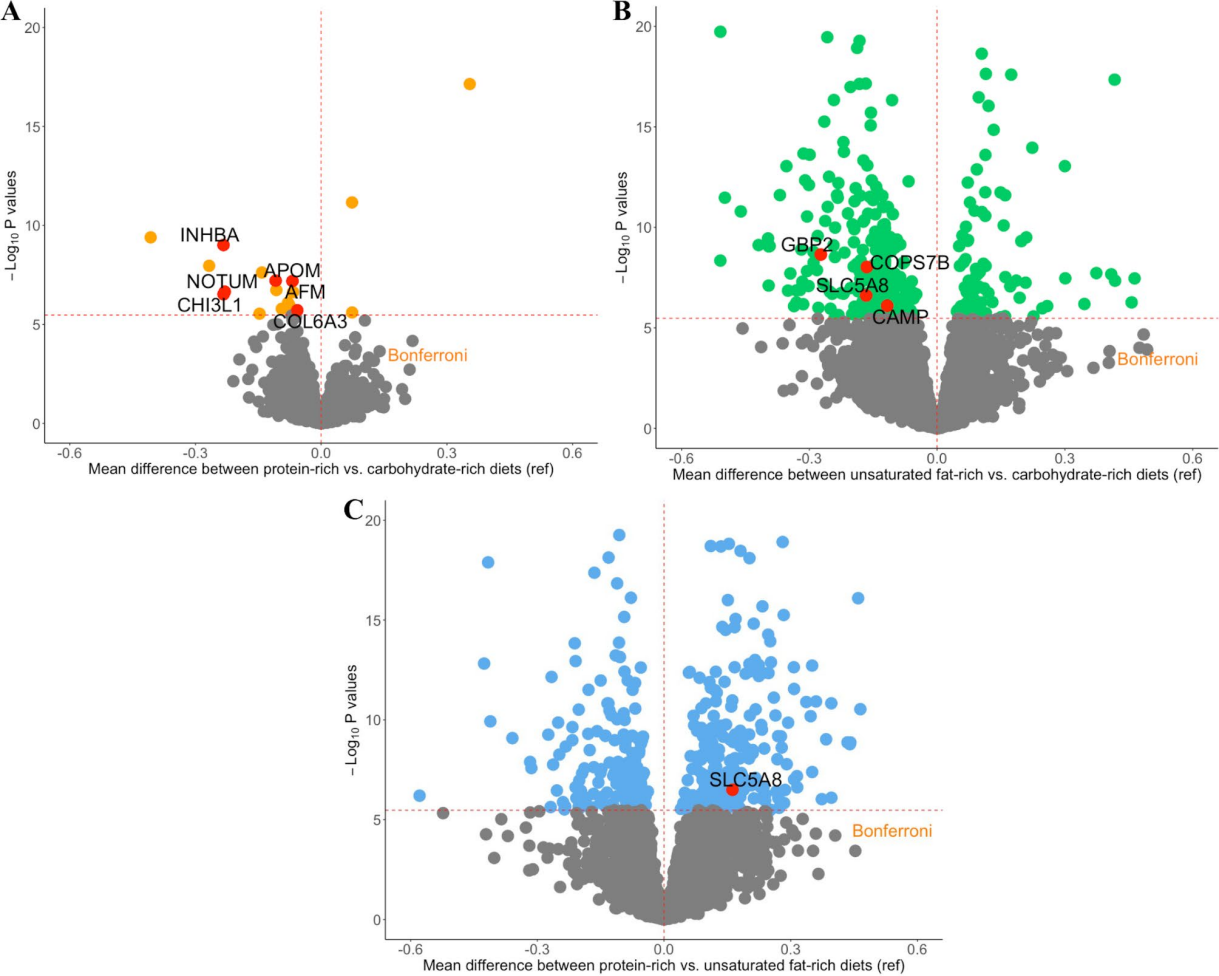
Volcano plots of mean differences and *P*-values for the association between individual plasma proteins and 3 diet comparisons: **(A)** protein-rich vs. carbohydrate-rich (reference), **(B)** unsaturated fat-rich vs. carbohydrate-rich (reference), and **(C)** protein-rich vs. unsaturated fat-rich dietary patterns (reference). The dashed horizontal line represents the Bonferroni-adjusted threshold (0.05/4,958/3 diet comparisons = 3.36 × 10^− 6^) and the vertical dashed line is set at a mean difference of zero. Proteins located to the right of the vertical line indicates that the abundance of proteins were higher than the reference diet, and proteins located to the left of the vertical line indicates that the abundance of proteins were lower than the reference diet. Data points which are colored in red and are labeled represent the proteins that significantly mediate the association between each diet comparison and lipoproteins (Table 3; Table 4). AFM, afamin; APOM, apolipoprotein M, CAMP, cathelicidin antimicrobial peptide; CHI3L1, chitinase-3-like protein 1; COL6A3, collagen alpha-3(VI) chain; COPS7B, COP9 signalosome complex subunit 7b; GBP2, guanylate-binding protein 2; INHBA, inhibin beta A chain; NOTUM, palmitoleoyl-protein carboxylesterase NOTUM; ref, reference; SLC5A8, sodium-coupled monocarboxylate transporter 1

Of the 4,958 proteins identified, 497 were associated with at least one diet comparison [protein-rich vs. carbohydrate-rich = 18 (0.4%); unsaturated fat-rich vs. carbohydrate-rich = 335 (6.8%); protein-rich vs. unsaturated fat-rich dietary patterns = 398 (8.0%)] at the Bonferroni threshold of 3.36 × 10^− 6^ (Additional file 2: Table S3). Compared to the carbohydrate-rich dietary pattern, abundance of most proteins was significantly lower on the protein-rich dietary pattern (15 out of 18 proteins) (Fig. 1A) and unsaturated fat-rich dietary pattern (246 out of 335 proteins) (Fig. 1B). Compared to the unsaturated fat-rich dietary pattern, abundance of the majority of proteins (239 out of 398 proteins) was higher in the protein-rich dietary pattern (Fig. 1C).

**Table 3:** Association between differences in diet-related plasma proteins and differences in lipoprotein concentrations in the OmniHeart Trial^*a*^

			HDL-C (mmol/L)		Triglyceride (mmol/L)		non-HDL-C (mmol/L)		TC:HDL-C ratio	
Name of the protein	Uniprot ID	Entrezgene symbol	$\beta$	*P*-value	$\beta$	*P*-value	$\beta$	*P*-value	$\beta$	*P*-value
**Protein-rich vs. carbohydrate-rich dietary pattern (reference) (*n*** **= 6)**										
Apolipoprotein M	O95445	APOM	0.29	4.86 × 10^− 6^	—	—	—	—	—	—
Afamin	P43652	AFM	0.48	1.96 × 10^− 6^	—	—	—	—	—	—
Collagen alpha-3(VI) chain	P12111	COL6A3	—	—	1.30	2.53 × 10^− 4^	1.19	4.02 × 10^− 5^	—	—
Chitinase-3-like protein 1	P36222	CHI3L1	—	—	0.34	2.79 × 10^− 4^	—	—	—	—
Inhibin beta A chain	P08476	INHBA	—	—	0.44	1.20 × 10^− 4^	—	—	—	—
Palmitoleoyl-protein carboxylesterase NOTUM	Q6P988	NOTUM	—	—	0.38	6.46 × 10^− 5^	—	—	—	—
**Unsaturated fat-rich vs. carbohydrate-rich dietary pattern (reference) (*n*** **= 4)**										
Cathelicidin antimicrobial peptide	P49913	CAMP	—	—	0.86	4.96 × 10^− 7^	0.65	1.12 × 10^− 6^	—	—
Guanylate-binding protein 2	P32456	GBP2	—	—	—	—	—	—	0.36	1.14 × 10^− 5^
COP9 signalosome complex subunit 7b	Q9H9Q2	COPS7B	—	—	—	—	0.49	1.38 × 10^− 5^	—	—
Sodium-coupled monocarboxylate transporter 1	Q8N695	SLC5A8	—	—	—	—	—	—	0.61	7.89 × 10^− 8^
**Protein-rich vs. unsaturated fat-rich dietary pattern (reference) (*n*** **= 1)**										
Sodium-coupled monocarboxylate transporter 1	Q8N695	SLC5A8	-0.24	1.26 × 10^− 10^	—	—	—	—	—	—

^*a*^ All diet comparisons lowered high-density lipoprotein (HDL)-cholesterol (C), triglycerides, non-HDL-C and the ratio of total cholesterol and HDL-C. The unsaturated fat-rich vs. carbohydrate-rich dietary patterns increased HDL-C concentration, and the protein-rich vs. unsaturated fat-rich dietary patterns found no difference in non-HDL-C (Additional file 2: Table S1). $\beta$ coefficients and *P*-values were calculated from linear regression models which used differences in proteins (exposure) and differences in lipoproteins at the end of the intervention period for each diet comparison (response). For the protein-rich vs. carbohydrate-rich dietary patterns, we used the Bonferroni threshold of 5.56 × 10^− 4^ (0.05/18 diet-related proteins/5 lipoprotein outcomes [low-density lipoprotein-C, HDL-C, triglycerides, total cholesterol, non-HDL-C, ratio of total cholesterol to HDL-C]). For unsaturated fat-rich vs. carbohydrate-rich dietary patterns, we used 2.99 × 10^− 5^ (0.05/335 diet-related proteins/5 lipoprotein outcomes). For protein-rich vs. unsaturated fat-rich dietary patterns, we used 2.51 × 10^− 5^ (0.05/398 diet-related proteins/5 lipoprotein outcomes). Diet-related proteins significantly associated with lipoprotein outcomes that had statistically significant mediation are presented. No diet-related protein was significantly associated low-density lipoprotein-C

— indicates that there was no significant association between the diet-related protein and lipoprotein outcome

TC, total cholesterol

**Table 4:** Proportion of the association between diet and lipoprotein concentrations mediated by diet-related plasma proteins in the OmniHeart trial^*a*^

			HDL-C (mmol/L)		Triglyceride (mmol/L)		non-HDL-C (mmol/L)		TC:HDL-C ratio	
Name of the protein	Uniprot ID	Entrezgene symbol	% mediated	*P*	% mediated	*P*	% mediated	*P*	% mediated	*P*
**Protein-rich vs. carbohydrate-rich dietary pattern (reference) (*n*** **= 6)**										
Apolipoprotein M	O95445	APOM	97.6	0.01	—	—	—	—	—	—
Afamin	P43652	AFM	56.9	0.01	—	—	—	—	—	—
Collagen alpha-3(VI) chain	P12111	COL6A3	—	—	25.2	0.002	25.1	< 0.001	—	—
Chitinase-3-like protein 1	P36222	CHI3L1	—	—	20.6	0.004	—	—	—	—
Inhibin beta A chain	P08476	INHBA	—	—	36.0	< 0.001	—	—	—	—
Palmitoleoyl-protein carboxylesterase NOTUM	Q6P988	NOTUM	—	—	41.8	< 0.001	—	—	—	—
**Unsaturated fat-rich vs. carbohydrate-rich dietary pattern (reference) (*n*** **= 4)**										
Cathelicidin antimicrobial peptide	P49913	CAMP	—	—	68.9	0.02	43.6	< 0.001	—	—
Guanylate-binding protein 2	P32456	GBP2	—	—	—	—	—	—	70.1	0.002
COP9 signalosome complex subunit 7b	Q9H9Q2	COPS7B	—	—	—	—	33.4	< 0.001	—	—
Sodium-coupled monocarboxylate transporter 1	Q8N695	SLC5A8	—	—	—	—	—	—	96.3	0.004
**Protein-rich vs. unsaturated fat-rich dietary pattern (reference) (*n*** **= 1)**										
Sodium-coupled monocarboxylate transporter 1	Q8N695	SLC5A8	73.6	< 0.001	—	—	—	—	—	—

^*a*^ All diet comparisons lowered high-density lipoprotein (HDL)-cholesterol (C), triglycerides, non-HDL-C and the ratio of total cholesterol and HDL-C. The unsaturated fat-rich vs. carbohydrate-rich dietary patterns increased HDL-C concentrations, and the protein-rich vs. unsaturated fat-rich dietary patterns found no difference in non-HDL-C (Additional file 2: Table S1). Proportion mediated and *P*-values were calculated using causal mediation analysis. We fit two linear mixed models with random intercepts. The first model evaluated the association between each of the diet comparisons and proteins (mediator). The second model evaluated the association between each of the diet comparisons and lipoprotein outcomes, after controlling for proteins. Diet-related plasma proteins significantly associated with lipoprotein outcomes that had statistically significant mediation are presented. No diet-related protein was significantly associated low-density lipoprotein-C

— indicates that there was no significant association between the diet-related protein and lipoprotein outcome

No proteins were common to the 3 diet comparisons, but 242 proteins were common to 2 comparisons: (1) unsaturated fat-rich vs. carbohydrate-rich and (2) protein-rich vs. unsaturated fat-rich dietary patterns. These data suggest that the common proteins are related to the unsaturated fat-rich dietary pattern (Additional file 1: Fig. S5). Further, 12 proteins were common to the (1) protein-rich vs. carbohydrate-rich and (2) protein-rich vs. unsaturated fat-rich dietary patterns.

### Proteins and lipoproteins

After excluding a protein with no significant mediation (*n* = 1) and those which produced negative proportion mediated (*n* = 2), of the 497 diet-related proteins, we found 13 significant diet-lipoprotein associations. For all pairwise comparisons in dietary patterns, we found ten proteins (protein-rich vs. carbohydrate-rich = 6; unsaturated-fat rich vs. carbohydrate-rich = 4; protein-rich vs. unsaturated fat-rich dietary patterns = 1) which were significantly associated with at least one lipoprotein outcome (Table 3). No diet-related protein was significantly associated with LDL-C concentrations.

For the comparison between protein-rich and carbohydrate-rich dietary patterns, abundance of all 6 diet-related proteins was lower on the protein-rich dietary pattern (Additional file 2: Table S3; Fig. 1A), and these proteins were positively associated with several lipoproteins [apolipoprotein M (APOM) and afamin (AFM) and HDL-C; collagen alpha-3(VI) chain (COL6A3), chitinase-3-like protein 1 (CHI3L1), inhibin beta A chain (INHBA), and palmitoleoyl-protein carboxylesterase NOTUM (NOTUM) and triglycerides; and COL6A3 and non-HDL-C] (Table 3).

For the comparison between unsaturated fat-rich and protein-rich dietary patterns, protein abundance of cathelicidin antimicrobial peptide (CAMP), guanylate-binding protein 2 (GBP2), COP9 signalosome complex subunit 7b (COPS7B), and SLC5A8 was all lower on the unsaturated fat-rich dietary pattern (Additional file 2: Table S3; Fig. 1B). These proteins were positively associated with several lipoproteins (CAMP for triglycerides and non-HDL-C; GBP2 and SLC5A8 for the ratio of total cholesterol to HDL-C; COPS7B for non-HDL-C).

For the comparison between protein-rich and unsaturated fat-rich dietary patterns, abundance of SL5A8 was higher on the protein-rich dietary pattern (Additional file 2: Table S3; Fig. 1C) and this protein was inversely associated with HDL-C concentrations.

In sex-stratified analyses, the direction of the associations between all proteins and lipoprotein outcomes was consistent across men and women, and consistent with the overall analytic sample (Additional file 1: Table S2).

### Mediation of proteins for the association between diet and lipoproteins

The proportion of the effect of diets on lipoproteins that was mediated by proteins, individually, ranged from 21 to 98% (Table 4). For instance, APOM and AFM mediated 98% and 57% (*P*-value = 0.01 for both tests) of the association between protein-rich vs. carbohydrate-rich dietary patterns and HDL-C concentrations, respectively. COL6A3, CHI3L1, INHBA, and NOTUM mediated 21–42% of the association between the same diet comparison and triglycerides (*P*-value < 0.01 for all tests). For the association between unsaturated fat-rich vs. carbohydrate-rich dietary patterns, CAMP mediated 69% of the association with triglycerides and 44% of the association with non-HDL-C concentrations (P < 0.05 for both tests). GBP2 and SLC5A8 mediated a larger proportion of the association (range = 70–96%) with the ratio of total cholesterol to HDL-C, and COPS7B mediated 33% of the association with non-HDL-C concentrations (*P*-value < 0.01 for all tests). For the protein-rich vs. unsaturated fat-rich dietary patterns, SLC5A8 mediated 74% of the association with HDL-C concentrations.

### Validation of diet-related proteins and lipoprotein associations in the ARIC Study

Of the 13 diet-lipoprotein associations in the OmniHeart trial, 12 associations were validated in the ARIC Study at the Bonferroni threshold (0.05/13 associations = 3.85 × 10^− 3^) (Table 5). Per doubling of diet-related proteins were positively associated with most lipoprotein outcomes, except for SLC5A8 and HDL-C which had an inverse association. AFM was not significantly associated with HDL-C in the ARIC Study.

**Table 5:** Validation of 10 diet-related proteins and lipoprotein outcomes in the Atherosclerosis Risk in Communities (ARIC) Study^*a*^

			HDL-C (mmol/L)		Triglyceride (mmol/L)		non-HDL-C (mmol/L)		TC:HDL-C ratio	
Name of the protein	Uniprot ID	Entrezgene symbol	$\beta$	*P*-value	$\beta$	*P*-value	$\beta$	*P*-value	$\beta$	*P*-value
Apolipoprotein M	O95445	APOM	0.11	1.09 × 10^− 57^	—	—	—	—	—	—
Afamin	P43652	AFM	0.002	7.92 × 10^− 1^	—	—	—	—	—	—
Collagen alpha-3(VI) chain	P12111	COL6A3	—	—	0.13	2.65 × 10^− 3^	-0.30	2.01 × 10^− 11^	—	—
Chitinase-3-like protein 1	P36222	CHI3L1	—	—	0.09	4.90 × 10^− 35^	—	—	—	—
Inhibin beta A chain	P08476	INHBA	—	—	0.69	2.20 × 10^− 193^	—	—	—	—
Palmitoleoyl-protein carboxylesterase NOTUM	Q6P988	NOTUM	—	—	0.09	1.44 × 10^− 8^	—	—	—	—
Cathelicidin antimicrobial peptide	P49913	CAMP	—	—	0.38	1.27 × 10^− 137^	0.29	1.74 × 10^− 73^	—	—
Guanylate-binding protein 2	P32456	GBP2	—	—	—	—	—	—	6.10	1.01 × 10^− 46^
COP9 signalosome complex subunit 7b	Q9H9Q2	COPS7B	—	—	—	—	0.15	4.39 × 10^− 30^	—	—
Sodium-coupled monocarboxylate transporter 1	Q8N695	SLC5A8	-0.06	7.67 × 10^− 36^	—	—	—	—	4.48	7.03 × 10^− 21^

^*a*^ Ten diet-related proteins which were associated with lipoproteins and significantly mediated diet-lipoprotein associations were validated in the ARIC Study (N = 11,201). Multivariable linear regression models were used to study the cross-sectional association between diet-related log_2_-transformed proteins (exposure) and lipoprotein outcomes (response), adjusting for age, sex, race-study center, smoking status, body mass index, and kidney function (estimated glomerular filtration rate). All of these proteins, except for afamin, were significant at the Bonferroni threshold (0.05/13 diet-lipoprotein associations = 3.85 × 10^− 3^)

— indicates that validation analyses were not conducted for the given protein and lipoprotein outcome in the ARIC Study, since the associations were not significant in the OmniHeart Trial

## Discussion

In this cross-over, randomized, controlled feeding trial which compared healthy dietary patterns varying in the proportion of macronutrient, we conducted 3 pairwise comparisons: (a) protein-rich vs. carbohydrate-rich, (b) unsaturated fat-rich vs. carbohydrate-rich, and (c) protein-rich and unsaturated fat-rich dietary patterns. We identified 497 proteins associated with at least one of these diet comparisons, after accounting for multiple testing. Of the 497 diet-related proteins, 10 proteins significantly mediated the association between at least one diet comparison and serum lipoproteins (a total of 13 associations), with a range of proportion mediated from 21 to 98%. Of the 13 diet-lipoprotein associations, 12 associations were validated in a large observational study. These associations represent modifiable pathways underlying diet and lipoproteins.

Our study replicated several proteins that were previously reported as proteomic markers of healthy dietary patterns. In Sweden, two population-based cohorts examined proteins associated with a healthy dietary pattern (high in fruits, vegetables, nuts, and fish), derived using a principal component analysis [10]. Matrix metalloproteinase (MMP)-7 was negatively associated with their data-derived healthy dietary pattern. In our study, we found 2 MMP proteins (MMP-14 and MMP-19) were associated with protein-rich vs. unsaturated fat-rich dietary patterns. Our study also replicated CVD-related proteins, cystatin C and angiopoietin-related protein 3 (ANGPTL3), which were inversely associated with diet quality scores in the Framingham Heart Study composed mostly of European Americans [7].

Compared to the carbohydrate-rich dietary pattern, the abundance of APOM was lower on the protein-rich dietary pattern, and APOM mediated the association with HDL-C concentrations. SLC5A8 is another protein which mediated the association between unsaturated fat-rich vs. carbohydrate-rich dietary patterns and the ratio of total cholesterol to HDL-C. APOM, secreted by the liver, is often bound to HDL in circulation, and was associated with reduced atherosclerosis in mouse models [20]. Carbohydrates have been shown to increase fractional catabolic rate of HDL proteins, specifically APOM-containing HDL particles [21]. Our results add to these findings that proteins, in addition to carbohydrates, may play a role in removing HDL in circulation. SLC5A8 is a membrane transporter of short-chain fatty acids [22]. In plasma, SLC5A8 may be an extracellular vesicle, but the role of plasma SLC5A8 in relation to diet and the ratio of total cholesterol to HDL remains unclear.

CHI3L1, INHBA, COL6A3 and NOTUM were another set of proteins with lower abundance in protein-rich vs. carbohydrate-rich dietary patterns and which mediated the associations with triglycerides. All of these proteins implicate that partial replacement of carbohydrate with protein may result in lower inflammation and lipid accumulation. CHI3L1, also known as YKL-40, is a glycoprotein produced by lipid-laden macrophages in the vessel wall and is expressed in epicardial adipose tissue among individuals with atrial fibrillation [23, 24]. YKL-40 is considered proinflammatory and has atherosclerotic effects [25]. Consistent with our findings, observational studies in the general population reported that circulating levels of YKL-40 are strongly associated with elevated concentrations of triglyceride and vascular diseases [26, 27]. In a secondary analysis of the OmniHeart trial, compared to the baseline, the 3 diets reduced high-sensitive C-reactive protein measured by nephelometric assay, but not between diets [28]. Our proteomics analysis, which included a large number of inflammatory proteins, suggests that the protein-rich dietary pattern reduced inflammation compared to the carbohydrate-rich dietary pattern. Similar to CHI3LI, INHBA and particularly COL6A3 are abundantly expressed in adipose tissue [29, 30]. One in vivo study found that COL6A3 expression in human adipocytes was positively correlated with serum triglycerides, BMI, and large adipocytes [31]. NOTUM is known to inhibit Wnt signaling by binding to Wnts [32]. Recently, it has been shown that Wnt signaling is essential for lipogenesis in adipocytes, and inhibition of this pathway led to downregulation of de novo lipogenesis enzymes in adipocytes [33].

Compared to the carbohydrate-rich dietary pattern, abundance of plasma protein COPS7B was lower in the unsaturated fat-rich dietary pattern, and COPS7B mediated the association with non-HDL-C concentrations. COPS7B is a component of the COP9 signalosome complex (CSN). CSN performs a variety of functions, such as regulation of ubiquitylation of other protein complexes [34]. It has been reported that CSN is involved in ubiquitylation of ATP-binding cassette transporter 1 (ABCA1), and increases ABCA1 stabilization and promotes cholesterol efflux [35]. Cholesterol efflux is a critical mechanism through which HDL removes excess cholesterol from peripheral tissues and transports it to the liver for excretion [36]. A greater intake of unsaturated fats, specifically polyunsaturated fats, has been demonstrated to increase cholesterol efflux [37, 38]. However, in our study, difference in PUFA intake was small (2% of total energy) whereas difference in MUFA intake was greater (10% of total energy) between carbohydrate-rich and unsaturated fat-rich dietary patterns. To our knowledge, no experimental evidence investigated the mechanisms through which replacing carbohydrate with PUFA or MUFA lowers non-HDL-C concentrations, which warrants confirmation in future studies [39].

GBP2 and CAMP (also known as LL-37) were inversely associated with unsaturated fat-rich vs. carbohydrate-rich dietary patterns and associated with adverse lipoprotein outcomes (triglycerides, non-HDL-C, and the ratio of total cholesterol to HDL-C). Consistent with the direction of the association in our study, plasma concentrations of LL-37 were positively associated with triglycerides and inversely associated with HDL-C in 133 adults with type 2 diabetes [40]. LL-37 is known to have antimicrobial activity [41]. LL-37 is produced in response to lipopolysaccharide (LPS) and is reported to neutralize bacterial LPS [42, 43]. LPS are endotoxins which are modulated by food intake [44]. Several studies found that greater intake of unsaturated fat can lower plasma LPS, and unsaturated fat intake, which lowers postprandial triglycerides, may also decrease LPS [45–47]. GBP, a family of GTPase, is another protein that mediates a range of innate immune functions, such as providing protection against bacteria [41]. Taken together, LL-37 and GBP2 suggests that it may be important to consider the role of gut microbiota for the association between diet and lipoprotein concentrations. Differences in macronutrient intake can influence the gut microbiota composition [48], which can have impact on immune-related proteins [49].

The present study has several strengths, including the use of data from a randomized, controlled feeding study and validation in a large observational study. The sample size of the trial was sufficient to detect differences in the primary outcomes (systolic blood pressure, LDL-C), and identify small differences in the relative abundance of proteins. Considering high adherence to each diet intervention [3], proteins that we identified likely reflect the true biology resulting from consumption of these healthy dietary patterns. Body weight was maintained constant in the OmniHeart trial, thereby minimizing the possibility of the impact of weight loss on lipoprotein profile. The OmniHeart trial and the ARIC study included black and white men and women, thus the findings are broadly generalizable.

In terms of limitations, it is important to note that the diet intervention period was relatively short (6 weeks), thus may not reflect long-term intake of the dietary patterns. We were not able to validate associations between the diets compared in the OmniHeart trial and significant proteins or to validate these diets and hard clinical outcomes in the ARIC Study. It is difficult to model isocaloric manipulation of macronutrient intake within the context of a healthy dietary pattern in an observational study, given the unique design of the OmniHeart trial. However, it is encouraging that most associations between diet-related proteins and lipoprotein outcomes validated in the ARIC study, which increases confidence that these associations may be true associations. In the OmniHeart trial, plasma proteins and serum lipoprotein outcomes were assessed at the same time (end of each intervention period). Proteomic profiling was not conducted at baseline, and blood specimens were not collected at the end of each washout period or at the beginning of each intervention period. Therefore, temporality could not be established between the mediator and the outcome. However, we applied strict criteria to select proteins as mediators (e.g., Bonferroni threshold, diet-related proteins that are significantly associated with serum lipoproteins) and excluded a few proteins with inconsistent direction of association for the mediated and direct effect. Although stored at -70 °C and never thawed, it is possible that there may have been degradation of proteins, because biospecimens were collected > 10 years ago. However, we expect any degradation to be non-differential by dietary patterns. Lastly, the SomaScan platform provides only relative quantification of proteins, and we did not have data on absolute protein concentrations.

## Conclusions

In conclusion, large-scale proteomics conducted in a randomized controlled feeding trial identified proteins which may be involved in the effect of healthy dietary patterns on lipoprotein outcomes. We validated most of the diet-related proteins and lipoprotein associations in an observational study. These proteins may be diet modifiable mechanisms which could be targeted to reduce CVD risk.

## Electronic supplementary material

Below is the link to the electronic supplementary material.

Additional file 1 (docx format): supplemental data on study design and study population, and original OmniHeart Trial results. Figure S1. Study design of the OmniHeart trial. Figure S2. Flow diagram of study participants in the OmniHeart proteomics study. Figure S3. Flow diagram of study participants in the ARIC Study. Figure S4. Proteins overlapping across 3 diet comparisons: (1) protein-rich vs. carbohydrate-rich (reference), (2) unsaturated fat-rich vs. carbohydrate-rich (reference), and (3) protein-rich vs. unsaturated fat-rich dietary patterns (reference). Table S1 Effect of dietary patterns which vary in macronutrients and lipoproteins. Table S2. Association between differences in diet-related plasma proteins and differences in lipoprotein concentrations in the OmniHeart Trial, stratified by sex.

Additional file 2 (xls format): Table S3. proteins significantly different for 3 diet comparisons in the OmniHeart Trial.

## Data Availability

Original data, codebook, and the full protocol of the OmniHeart Trial are available upon request pending application and approval from the National Heart, Lung, and Blood Institute Biologic Specimen and Data Repository Information Coordinating Center: https://biolincc.nhlbi.nih.gov/studies/omniheart/?q=Omni Heart. Sharing of proteomics data follow an existing data sharing plan. Analytic codes that support the results of the present study are available from the corresponding author (Dr. Casey M. Rebholz crebhol1@jhu.edu) upon reasonable request. A list of proteins associated with dietary patterns has been uploaded as additional files.

## List of Abbreviations

AFM: Afamin
APOM: Apolipoprotein M
CAMP: Cathelicidin antimicrobial peptide
CHI3L1: Chitinase-3-like protein 1
COL6A3: Collagen alpha-3(VI) chain
COPS7B: COP9 signalosome complex subunit 7b
CSN: COP9 signalosome complex
CVD: Cardiovascular disease
DASH: Dietary Approaches to Stop Hypertension
eGFR: Estimated glomerular filtration rate
GBP2: Guanylate-binding protein 2
HDL: High-density lipoprotein
INHBA: Inhibin beta A chain
LDL: Low-density lipoprotein
NOTUM: Palmitoleoyl-protein carboxylesterase NOTUM
OmniHeart: The Optimal Macronutrient Intake Trial to Prevent Heart Disease
SLC5A8: Sodium-coupled monocarboxylate transporter 1
SOMAmer: Slow Off-rate Modified Aptamer
