# Supplementary material for: Associations of circulating proteins with lipoprotein profiles: proteomic analyses from the OmniHeart randomized trial and the Atherosclerosis Risk in Communities (ARIC) Study
Source: Clin Proteomics. 2023 Jul 3;20:27. doi: 10.1186/s12014-023-09416-x (PMC10316599; doi:10.1186/s12014-023-09416-x)
Supplement: Supplementary file 1 — Additional file 1 (docx format): supplemental data on study design and study population, and original OmniHeart Trial results. Figure S1. Study design of the OmniHeart trial. Figure S2. Flow diagram of study participants in the OmniHeart proteomics study. Figure S3. Flow diagram of study participants in the ARIC Study. Figure S4. Proteins overlapping across 3 diet comparisons: (1) protein-rich vs. carbohydrate-rich (reference), (2) unsaturated fat-rich vs. carbohydrate-rich (reference), and (3) protein-rich vs. unsaturated fat-rich dietary patterns (reference). Table S1 Effect of dietary patterns which vary in macronutrients and lipoproteins. Table S2. Association between differences in diet-related plasma proteins and differences in lipoprotein concentrations in the OmniHeart Trial, stratified by sex. [file 12014_2023_9416_MOESM1_ESM.docx]

**Additional file 1**

**3 diet interventions in random order**

**Control Diet**

**Run-in:**

**(6-day)**

Participants ate their own foods

**Intervention**

**(Three 6-week periods, cross-over trial, 2 to 4 weeks of wash-out period between each diet intervention)** Participants ate their own foods during the wash-out periods

**N = 164**

***Unsaturated fat-rich dietary pattern***

*48% Carbohydrate*

*15% Protein*

*37% Fat*

***Protein-rich***

***dietary pattern***

*48% Carbohydrate*

*25% Protein*

*27% Fat*

***Carbohydrate-rich***

***dietary pattern***

*58% Carbohydrate*

*15% Protein*

*27% Fat*

**
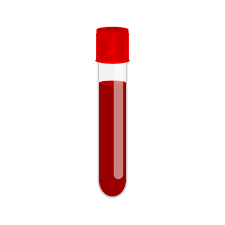

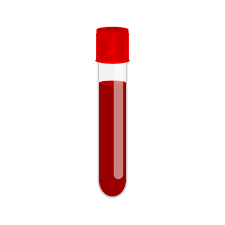

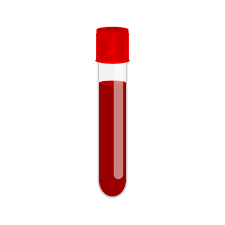
**

- **Protein profiling**
- **Lipoprotein outcomes**
- **Protein profiling**
- **Lipoprotein outcomes**
- **Protein profiling**
- **Lipoprotein outcomes**

**Figure S1**. Study design of the OmniHeart trial. Protein profiling and 5 lipoprotein outcomes (lipoprotein (LDL)-cholesterol (C), high-density lipoprotein (HDL)-C, triglycerides, and non-HDL-C, and the ratio of total cholesterol to HDL) were assessed at the end of each diet intervention period.

OmniHeart trial

(N=164)

Participants with no

plasma specimen

(n=3)

Participants missing proteomics data for at least one of the diet interventions

(n=8)

Participants with a flagged specimen for at least one of the diet interventions

(n=13)

Final analytic sample

(N=140)

**Figure S2.** Flow diagram of study participants in the OmniHeart proteomics study

Participants who attended visit 2

(N=14,348)

Participants excluded due to small numbers

[non-black or non-white participants (n=42),

Blacks in Minneapolis, Minnesota; or Blacks in Washington County, Maryland

(n=49)]

Participants with no proteomics data

(n=2,537)

Participants with incomplete lipoprotein concentrations

(n=479)

Participants with missing covariates^1^

(n=40)

Final analytic sample

(N=11,201)

**Figure S3.** Flow diagram of study participants in the ARIC Study

^1^ Covariates include: age (n=0), sex (n=0), race (n=0), study center (n=0), smoking status (n=19), BMI (n=18), and kidney function (n=3)

**Mediators – OmniHeart trial**

*Differences in plasma proteins for 3 diet intervention comparisons*

**Validation in the ARIC Study**

*Plasma proteins and 5 lipid outcomes*

Indirect effect quantified by proportion (%) mediated

Direct effect

**Exposure – OmniHeart trial**

*3 diet intervention comparisons*

1. Protein-rich vs. carbohydrate-rich
2. Unsaturated fat-rich vs. carbohydrate-rich
3. Protein-rich vs. unsaturated fat-rich

**Outcome – OmniHeart trial**

*Differences in 5 lipid outcomes for 3 diet intervention comparisons*

1. LDL-C
2. HDL-C
3. Triglycerides
4. Non-HDL
5. The ratio of total cholesterol to HDL

**Figure S4.** Overview of the mediation analysis for the association between dietary patterns and serum lipoprotein concentrations. Indirect effect represents effect of dietary patterns on lipoproteins that is mediated by plasma proteins. Direct effect represents effect of dietary patterns on lipoproteins with no mediation. Proportion (%) mediated is calculated using indirect effect divided by the sum of direct and indirect effects. ARIC Study, Atherosclerosis Risk in Communities Study; HDL-C, high-density lipoprotein-cholesterol; LDL, low-density lipoprotein-cholesterol


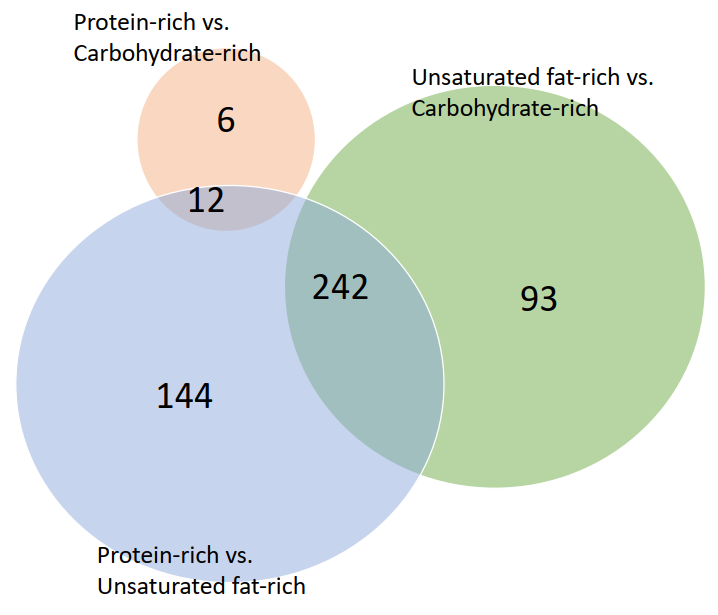


**Figure S5**. Proteins overlapping across 3 diet comparisons: 1) protein-rich vs. carbohydrate-rich (reference), 2) unsaturated fat-rich vs. carbohydrate-rich (reference), and 3) protein-rich vs. unsaturated fat-rich dietary patterns (reference)

**Supplemental Table S1**. Effect of dietary patterns which vary in macronutrients and lipoproteins

|  | Protein-rich vs. carbohydrate-rich dietary patterns (reference) | | Unsaturated fat-rich vs. carbohydrate-rich dietary patterns (reference) | | Protein-rich vs. unsaturated fat-rich dietary patterns (reference) | |
| --- | --- | --- | --- | --- | --- | --- |
|  | Mean | *P*-value | Mean | *P*-value | Mean | *P*-value |
|  |  |  |  |  |  |  |
| LDL-C (mg/dL) | -3.3 | 0.01 | -1.5 | 0.22 | -1.5 | 0.24 |
|  |  |  |  |  |  |  |
| HDL-C (mg/dL) | -1.3 | 0.02 | 1.1 | 0.03 | -2.3 | <0.001 |
|  |  |  |  |  |  |  |
| Triglyceride (mg/dL) | -15.7 | <0.001 | -9.6 | 0.02 | -7.1 | 0.03 |
|  |  |  |  |  |  |  |
| Non-HDL-C (mg/dL) | -6.5 | <0.001 | -4.2 | 0.002 | -2.6 | 0.054 |
|  |  |  |  |  |  |  |
| TC: HDL-C ratio^1^ | -0.06 | 0.24 | -0.14 | 0.002 | 0.08 | 0.11 |

^1^ Effect of dietary patterns on TC: HDL-C ratio was not reported in the main study findings paper. Therefore, TC: HDL-C ratio was calculated based on the analytic sample of the present study (N=140).

HDL-C, high-density lipoprotein-cholesterol; LDL-C, low-density lipoprotein-cholesterol; TC, total cholesterol.

**Supplemental Table S2.** Association between differences in diet-related plasma proteins and differences in lipoprotein concentrations in the OmniHeart Trial, stratified by sex*^a^*

|  |  | Men (N=73) | | | | | | | | Women (N=67) | | | | | | | |
| --- | --- | --- | --- | --- | --- | --- | --- | --- | --- | --- | --- | --- | --- | --- | --- | --- | --- |
|  |  | HDL-C  (mmol/L) | | Triglyceride (mmol/L) | | non-HDL-C (mmol/L) | | TC:HDL-C ratio | | HDL-C  (mmol/L) | | Triglyceride (mmol/L) | | non-HDL-C (mmol/L) | | TC:HDL-C ratio | |
| Name of the protein | Uniprot ID | $\beta$ | *P*-value | $\beta$ | *P*-value | $\beta$ | *P*-value | $\beta$ | *P*-value | $\beta$ | *P*-value | $\beta$ | *P*-value | $\beta$ | *P*-value | $\beta$ | *P*-value |
| **Protein-rich vs carbohydrate-rich dietary pattern (reference) (*n*=6)** | | | | | | | | | | | | | | | | | |
| Apolipoprotein M | O95445 | 0.25 | 9.59E-05 | — | — | — | — | — | — | 0.43 | 1.97E-03 | — | — | — | — | — | — |
| Afamin | P43652 | 0.19 | 8.19E-02 | — | — | — | — | — | — | 0.87 | 6.34E-07 | — | — | — | — | — | — |
| Collagen alpha-3(VI) chain | P12111 | — | — | 2.02 | 6.98E-05 | 1.36 | 7.28E-04 | — | — | — | — | 0.29 | 5.24E-01 | 0.98 | 2.35E-02 | — | — |
| Chitinase-3-like protein 1 | P36222 | — | — | 0.39 | 4.93E-03 | — | — | — | — | — | — | 0.28 | 2.45E-02 | — | — | — | — |
| Inhibin beta A chain | P08476 | — | — | 0.50 | 1.47E-03 | — | — | — | — | — | — | 0.31 | 5.83E-02 | — | — | — | — |
| Palmitoleoyl-protein carboxylesterase NOTUM | Q6P988 | — | — | 0.49 | 9.18E-05 | — | — | — | — | — | — | 0.12 | 4.39E-01 | — | — | — | — |
| **Unsaturated fat-rich vs carbohydrate-rich dietary pattern (reference) (*n*=4)** | | | | | | | | | | | | | | | | | |
| Cathelicidin antimicrobial peptide | P49913 | — | — | 0.93 | 2.44E-04 | 0.90 | 1.24E-07 | — | — | — | — | 0.74 | 8.98E-04 | 0.29 | 1.73E-01 | — | — |
| Guanylate-binding protein 2 | P32456 | — | — | — | — | — | — | 0.44 | 2.14E-04 | — | — | — | — | — | — | 0.23 | 3.89E-02 |
| COP9 signalosome complex subunit 7b | Q9H9Q2 | — | — | — | — | 0.53 | 3.73E-04 | — | — | — | — | — | — | 0.43 | 1.53E-02 | — | — |
| Sodium-coupled monocarboxylate transporter 1 | Q8N695 | — | — | — | — | — | — | 0.73 | 1.33E-04 | — | — | — | — | — | — | 0.49 | 9.93E-05 |
| **Protein-rich vs. unsaturated fat-rich dietary pattern (reference) (*n*=1)** | | | | | | | | | | | | | | | | | |
| Sodium-coupled monocarboxylate transporter 1 | Q8N695 | -0.20 | 1.39E-06 | — | — | — | — | — | — | -0.28 | 1.15E-05 | — | — | — | — | — | — |

*^a^* All diet comparisons lowered high-density lipoprotein (HDL)-cholesterol (C), triglycerides, non-HDL-C and the ratio of total cholesterol and HDL-C. The unsaturated fat-rich vs. carbohydrate-rich dietary patterns increased HDL-C concentration, and the protein-rich vs. unsaturated fat-rich dietary patterns found no difference in non-HDL-C (**Additional file 2: Table S1**). $\beta$ coefficients and *P*-values were calculated from linear regression models which used differences in proteins (exposure) and differences in lipoproteins at the end of the intervention period for each diet comparison (response), stratified by sex. For the protein-rich vs. carbohydrate-rich dietary patterns, we used the Bonferroni threshold of 5.56 × 10^-4^ (0.05/18 diet-related proteins/5 lipoprotein outcomes [low-density lipoprotein-C, HDL-C, triglycerides, total cholesterol, non-HDL-C, ratio of total cholesterol to HDL-C]). For unsaturated fat-rich vs. carbohydrate-rich dietary patterns, we used 2.99 × 10^-5^ (0.05/335 diet-related proteins/5 lipoprotein outcomes). For protein-rich vs. unsaturated fat-rich dietary patterns, we used 2.51 × 10^-5^ (0.05/398 diet-related proteins/5 lipoprotein outcomes). Diet-related proteins significantly associated with lipoprotein outcomes that had statistically significant mediation are presented. No diet-related protein was significantly associated low-density lipoprotein-C.

— indicates that there was no significant association between the diet-related protein and lipoprotein outcome.

TC, total cholesterol
